# Preliminary study of BF/C2 on immune mechanism of grass carp against GCRV infection

**DOI:** 10.1186/s12864-024-10609-3

**Published:** 2024-07-24

**Authors:** Yuling Wei, Yu Xiao, Qiaolin Liu, Zongjun Du, Tiaoyi Xiao

**Affiliations:** 1 Hunan Engineering Technology Research Center of Featured Aquatic Resources Utilization, Hunan Agricultural University, Changsha, Hunan 410128 China; 2 College of Animal Science and Technology, Sichuan Agricultural University, Chengdu, 611130 China

**Keywords:** *BF/C2*, Grass carp, Kidney cell, Transcriptome, Immunity

## Abstract

*BF/C2* is a crucial molecule in the coagulation complement cascade pathway and plays a significant role in the immune response of grass carp through the classical, alternative, and lectin pathways during GCRV infection. In vivo experiments demonstrated that the mRNA expression levels of *BF/C2* (*A*, *B*) in grass carp positively correlated with GCRV viral replication at various stages of infection. Excessive inflammation leading to death coincided with peak levels of *BF/C2* (*A*, *B*) mRNA expression and GCRV viral replication. Correspondingly, *BF/C2* (*A*, *B*) recombinant protein, CIK cells and GCRV co-incubation experiments yielded similar findings. Therefore, 3 h (incubation period) and 9 h (death period) were selected as critical points for this study. Transcriptome sequencing analysis revealed significant differences in the expression of *BF/C2A* and *BF/C2B* during different stages of CIK infection with GCRV and compared to the blank control group (PBS). Specifically, the *BF/C2A*_3 and *BF/C2A*_9 groups exhibited 2729 and 2228 differentially expressed genes (DEGs), respectively, with 1436 upregulated and 1293 downregulated in the former, and 1324 upregulated and 904 downregulated in the latter. The *BF/C2B*_3 and *BF/C2B*_9 groups showed 2303 and 1547 DEGs, respectively, with 1368 upregulated and 935 downregulated in the former, and 818 upregulated and 729 downregulated in the latter. KEGG functional enrichment analysis of these DEGs identified shared pathways between *BF/C2A* and PBS groups at 3 and 9 h, including the C-type lectin receptor signaling pathway, protein processing in the endoplasmic reticulum, Toll-like receptor signaling pathway, Salmonella infection, apoptosis, tight junction, and adipocytokine signaling pathway. Additionally, the *BF/C2B* groups at 3 and 9 h shared pathways related to protein processing in the endoplasmic reticulum, glycolysis/gluconeogenesis, and biosynthesis of amino acids. The mRNA levels of these DEGs were validated in cellular models, confirming consistency with the sequencing results. In addition, the mRNA expression levels of these candidate genes (*mapk1*, *il1b*, *rela*, *nfkbiab*, *akt3a*, *hyou1*, *hsp90b1*, *dnajc3a* et al.) in the head kidney, kidney, liver and spleen of grass carp immune tissue were significantly different from those of the control group by *BF/C2* (*A*, *B*) protein injection in vivo. These candidate genes play an important role in the response of BF/C2 (A, B) to GCRV infection and it also further confirmed that *BF/C2* (*A*, *B*) of grass carp plays an important role in coping with GCRV infection.

**Supplementary Information:**

The online version contains supplementary material available at 10.1186/s12864-024-10609-3.

## Introduction

The complement system is a highly conserved part of the innate immune system, encompassing various membrane-bound and soluble components closely related to the adaptive immune system. It is involved in initiating the adaptive immune response and supports numerous host defense mechanisms, including chemotaxis, opsonization, induction of inflammatory responses, and the cleavage of microbes, apoptotic cells, and immune complexes [1]. The system consists of a complex and nuanced cascade of over 50 soluble and cell-binding proteins, primarily found in serum and cell membranes [2–4]. It activates through three main pathways (classical, alternative, and lectin) and regulates lysis through one pathway. Each pathway is initiated by distinct pathogen patterns and involves different recognition molecules [5–7]. The classical pathway (CP) is triggered by antigen–antibody complexes or C-reactive protein, the lectin pathway (LP) detects mannose on bacterial surfaces, and the alternative pathway (AP) functions continuously at low levels through spontaneous *C3* hydrolysis [5, 8]. Though driven by various mechanisms, all pathways converge on the cleavage of key proteins *C3* and *C5*, activating the membrane attack complex (MAC) and inducing the cleavage of target cells [8]. Numerous studies have highlighted the indispensable role of the complement system in defending against pathogens [9].

In this system, *BF/C2* is an essential molecule. In mammals, *BF* and *C2* derive from different pathways: *BF* is crucial in the alternative pathway, while *C2* is important in both the classical and lectin pathways. In fish, these components are not distinguishable, leading to the general use of the term *BF/C2* [10]. Specifically, in grass carp, *BF/C2* encompasses *BF/C2A* and *BF/C2B*, which fold into three globular domains similar to those of *BF* and *C2* in humans and mice. The correlation between *BF/C2A*, *BF/C2B*, and *BF* and *C2* in fish mirrors that found in bony fish, cartilaginous sharks, and jawless lampreys [11, 12]. The specific role of *BF/C2* in the fish complement system remains a key focus of ongoing research. Whether *BF/C2* plays the same role in fish and mammals is also the focus of current research.

In mammals, *BF* and *C2* have been identified as interferon-stimulating genes [13–15]. Clinical studies using interferon treatments for human viral diseases demonstrated significant induction of *BF* and *C2* expression by interferon. Subsequent research indicated that both α-interferon and γ-interferon could stimulate the production of *BF* and *C2*, associated with the presence of interferon-stimulated response elements (ISRE) and γ-interferon activation sequence (GAS) on the promoters of the *BF* and *C2* genes [14, 15]. These elements on the promoter can be recognized and bound by the transcription factors *IRFs* and *STATs*, activated by interferon signals, thereby activating the promoter and enhancing the transcriptional expression of *BF* and *C2* [14, 15]. Hence, *BF* and *C2* are considered typical interferon-stimulating genes. Studies have verified that the complement proteins *BF* and *C2* predominantly exert an antiviral effect by activating the complement system. Knockout experiments in mice, lacking the *BF* or *C2* gene, showed that these mice struggled to combat infections such as influenza A virus, West Nile virus, and poxvirus, indicating a reduced activation of *C3* and the complement system, increased viral proliferation, and higher susceptibility and mortality [14, 15]. Moreover, the antiviral response of the *BF* and *C2* genes varies among species. For instance, in response to dengue virus infection, the expression level of the mouse *BF* gene was higher than that in humans, resulting in a more pronounced activation of *C3* and the complement system and, consequently, enhanced disease resistance in mice. This difference is attributed to the greater number of ISRE and GAS sites on the mouse BF gene promoter compared to humans [14, 15]. Considering the sequential relationship between promoter activity, gene expression, and disease resistance, increasing evidence suggests that variations in the *BF* and *C2* promoters are closely linked to human resistance to viral diseases. Given this background, the role of *BF/C2* in grass carp, particularly whether a similar regulatory mechanism exists, remains an intriguing question for further investigation.

Grass carp (*Ctenopharyngodon idella*), the highest producing freshwater aquaculture species in our country, reached a production of 590.48 million tons in 2022 [16]. However, the aquaculture industry faces significant challenges due to hemorrhagic disease caused by Grass carp reovirus (GCRV), with the low immunity of first-year grass carp being a primary factor in the high mortality rates among young fish induced by GCRV [17]. In 2012, our team conducted a transcriptomic analysis of the spleen of first-year grass carp before and after GCRV infection. We found that the differences in *BF/C2* were particularly pronounced within the complement-coagulation cascade pathway, which exhibited the most significant changes [18]. Additionally, using modern molecular-assisted resistance breeding alongside traditional techniques, our team assessed the activity level of the *BF/C2* complement protein in the plasma of grass carp parental stocks from the Xiangjiang and Yangtze Rivers using ELISA. The results indicated that *BF/C2* complement protein levels were normally distributed within the grass carp population. Furthermore, in 2014, our research group immunized female grass carp with a GCRV attenuated vaccine, resulting in offspring with significantly enhanced resistance to GCRV [19, 20]. It was observed that the serum of offspring from highly immune-resistant mothers exhibited elevated expressions of complement proteins (*BF/C2*, *C3*, etc.) [19, 20]. These findings suggest that *BF/C2* may play a crucial role in the resistance of grass carp to GCRV infection. Consequently, this study undertook a transcriptomic analysis of *BF/C2* (*A*, *B*) proteins (with PBS as a control) during different stages of GCRV infection in CIK cells, aiming to elucidate the immune mechanism of *BF/C2* (*A*, *B*) in response to GCRV infection in grass carp.

## Material and method

### *BF/C2*(*A*, *B*) protein-GCRV-CIK cell lines co-incubation experiment

To examine the impact of GCRV on *C. idellus* kidney cells (CIK) following incubation with *BF/C2*(*A*, *B*) protein, CIK cell lines established by our research group were utilized (They were cultured in M199 medium containing 1% penicillin–streptomycin and 10% fetal bovine serum at 28 ℃ in an incubator containing 5% CO2 and saturated humidity). Initially, well-cultured CIK cells were seeded in six-well plates and allowed to grow overnight until they completely covered the bottom of the plates. Subsequently, *BF/C2*(*A*, *B*) protein [21] was added at a final concentration of 380 ng/ml. After two hours of incubation, GCRV (strain: 1.58 × 10^4 TCID50/mL, GCRV-873 strain) was introduced to the cells. Samples were then collected at various time points: 1 h, 3 h, 6 h, 9 h, 12 h, and 24 h post-infection. Gene expression level of *vp7* (GCRV) were analyzed by quantitative PCR (qPCR).

### The experiment with grass carp infected with GCRV

Grass carps (5–7 cm in body length) were sourced from Huarong County, Hunan Province, China. The fish underwent a one-week acclimation period in recirculating freshwater tanks maintained at 28 °C and were fed a commercial diet equivalent to 3% of their body weight twice daily before any experimental procedures. The animal experiments were conducted in accordance with the guidelines approved by the Animal Care and Use Committee of Hunan Agricultural University (Changsha, China; Approval Code: 201,903,295; Approval Date: September 13, 2019). A total of 100 grass carps were used for the GCRV challenge experiment and were randomly allocated into two groups. Fish in the experimental group were exposed to the GCRV virus (a type II virus (*Huan1307*) donated by the Pearl River Fisheries Research Institute, Chinese Academy of Fishery Sciences) by immersion for 10 min before being returned to the circulation tanks, while the others served as the control group. Grass carps were randomly sampled (five individuals per time point) at five different stages as identified in prior research, including the incubation period (12 h post GCRV challenge, prior to the onset of symptoms), onset period (when symptoms began to emerge), death period (when grass carps started dying), recovering period (when grass carps began to recover), and restored period (when grass carps fully recovered and symptoms had disappeared). After anaesthesia with MS-222 (25 mg/L) (Sigma Aldrich Co., St. Louis, USA) prior to sample collection, liver tissue from each sample was collected and stored at -80 °C until RNA was extracted.

### Sample preparation

In this study, CIK cell lines established by our research group were utilized. Initially, well-cultured CIK cells were seeded in six-well plates and allowed to grow overnight until they completely covered the bottom of the plates. Subsequently, *BF/C2* (*A*,*B*) protein was added to achieve a final concentration of 380 ng/ml. After an incubation period of 2 h, GCRV was introduced to the wells. The cells were then incubated for additional periods of 3 h and 9 h, after which they were harvested using Trizol reagent and promptly stored at -80 °C.

### Library construction, and high-throughput sequencing

Total RNA of CIK samples was extracted and evaluated for purity and quantity. Quality assessment was performed according to the RNA quality assessment criteria. After meeting the assessment conditions, RNA libraries were constructed and sequenced, producing 150 bp-long paired-end reads. The above sequencing process and analysis were performed by Novogene Bioinformatics Technology Co. Ltd. (Beijing, China). Please refer to [22] for sequencing standards and specific procedures.

### RNA sequencing analysis

Raw RNA data were obtained in FASTQ format and processed using fastp (Version 20.1, length required 50). This involved the removal of reads containing poly-N sequences and low-quality reads to produce clean reads. Adaptor sequences and low-quality sections were also removed before the clean reads were assembled into expressed sequence tag clusters (contigs). These contigs were then subjected to de novo assembly into transcripts using Trinity (Version 2.4, seqType fq, SS_lib_type RF) via the paired-end method. The longest transcript from each assembly was selected as a unigene based on similarity and length for subsequent analyses. Functional annotation of the unigenes was performed using the Swiss-Prot database with the diamond tool, applying a threshold of e < 1 × 10^−5^. Proteins that showed the highest similarity to the unigenes were used to assign functional annotations. Additionally, the unigenes were mapped against the Kyoto Encyclopedia of Genes and Genomes (KEGG) database to annotate their potential involvement in various metabolic pathways. Each unigene was quantified and its expression level was calculated, and then differential expression unigenes (DEGs) among different groups were identified [22]. This software calculated differences using a negative binomial distribution test to evaluate the significance. Hierarchical cluster analysis of DEGs was conducted using R (version 3.2.0) to visualize the expression patterns of unigenes across different experimental groups and samples. KEGG pathway enrichment analysis of the DEGs was also performed in R based on the hypergeometric distribution, helping to identify significantly impacted metabolic pathways.

### qPCR analysis

Total RNA was extracted from the samples with an RNA extraction reagent (e.zn.a. ®Total RNA Kit II (Omega, Norcross, GA, USA)) and RNA quality was determined. cDNA synthesis was conducted utilizing the RevertAid™ First Strand cDNA Synthesis Kit (Thermo Fisher Scientific, Waltham, MA, USA), adhering to the manufacturer's protocol. The cDNA was used as a template for Quantitative PCR (qPCR). The qPCR process was executed on the CFX96 Touch™ Real-Time PCR Detection System (Bio-Rad, Hercules, CA, USA). The comparative threshold cycle method (2^−ΔΔCT^) was used to analyze the expression levels of target genes with* β*-actin as the reference gene. Each experiment involved three biological replicates. Table 1 lists the primers used in this study.

**Table 1:** Nucleotide sequences of the primers

Abbreviations	Primer sequence (5′ − 3′)	GenBank
*nfkb1*	F: GCCATTCACCTACCATCC	XM_051918332.1
	R: TCTGTATCACTGTCGCTATC	
*nfkbiaa*	F: GCTCCATTCTCACCTTCC	XM_051876441.1
	R: GTCGTCATACATACAGTCATC	
*nfkbiab*	F: TACAGAACAACCAGAGACAG	XM_051868091.1
	R: TCCACCAGAGAAGCATCA	
*dnajc3a*	F: GGAAGAATGGTGGTGTTGA	XM_051905660.1
	R: TGCTGAGGTCTGGTAGTG	
*dnajc3b*	F: GCATCACGACTCACTTAATC	XM_051901743.1
	R: CGCATCACAGACTCATACT	
*dnajb11*	F: GAAGTAGTCTGTGATGAATGC	XM_051906087.1
	R: TCTGAGATGGTGCCTGTT	
*derl3*	F: AACAGCGTCACTCAAGAG	XM_051903694.1
	R: AAGAACACCACCGAACAG	
*mapk1*	F: ATTACCTGCTGTCACTTCC	XM_051895297.1
	R: CTCCTCCACCTCAATCCT	
*mapk9*	F: TCACACCACAGAAGTCATTA	XM_051876678.1
	R: GTTCCTTACAGTCTCCATCA	
*mapkapk3*	F: AGACATCAAGCCAGAGAAC	XM_051912797.1
	R: CCAGAGACCACATATCACAT	
*hspa4a*	F: TGGAGGAAGAGAAGGTGTT	XM_051878238.1
	R: CTGCTGTAGTGTCGTTCAT	
*hspa5*	F: CCGCATCACTCCATCATAT	XM_051893937.1
	R: GTTCTTCTCACCATCCTTCT	
*hsp90aa1.2*	F: GCTTCGCTACTACACATCT	XM_051874259.1
	R: ACCTTCTCAACCTTCTTCTC	
*hsp90b1*	F: TGAGGTTGAGGAAGAGGAT	XM_051890068.1
	R: CAGCAGTGAAGTGAATGTG	
*akt3a*	F: CACCTCACAGATAGACAACA	XM_051917906.1
	R: TACTCCATCACGAAGCATAG	
*il1b*	F: GCTGATTCTGATGAGATGGA	XM_051908150.1
	R: GGTCTTGCTGGTCTTATAGTA	
*rela*	F: AACCAAGAACCAGCCATAC	XM_051900068.1
	R: CACCTCAATGTCCTCCTTC	
*hmgcs1*	F: TGGCTCTGCTCTGGATAA	XM_051909652.1
	R: AGGTCTGTCATCGTTCATAG	
*hyou1*	F: AGGCTTCTAACTGGATGGA	XM_051910945.1
	R: GGAGGTGCTGTTCTTGTC	
*herpud1*	F: ACCATCTGTGACCTCCTTA	XM_051869537.1
	R: TGTCCTCCTCATCTTCCAT	
*cxcl8a*	F: TGAACACCTACAGCATCG	XM_051892498.1
	R: GCCACAGCAACAATAACAA	
*erol*	F: CTGACAGTGGAGATGATGG	XM_051868039.1
	R: GGAAGTAGAGGTTGCGTAG	
*atf4*	F: CCATCACCTTCCATCCTTC	XM_051886449.1
	R: TTCTTCTCAACCACAACCTT	
*c3a- receptor-like*	F: CCTCTACACCATTACCATTATC	XM_051873504.1
	R: ACTTGAAGAAGCCATTGAAC	
*c1r*	F: ATGGACAGGAGACAACAAC	XM_051865200.1
	R: TGATGGCACAGTATGGAAG	
*c1q*	F: TCAGCACGGTTACATACAA	XM_051886808.1
	R: GAATGAAGCCAGAGAAGGT	
*β-actin*	F: CCTTCTTGGGTATGGAATCTTG	XM_051886219.1
	R: AGAGTATTTACGCTCAGGTGGG	
*vp7*	F: ACCACCAACTTTGATCACGCTGAG	AF403396.1
	R: AGCGTGGGAGTCTTGAATGGTCTT	
*Huan1307*	F: GTACAGCATTTGGCACGTCT R: TCCGCTGAATCGACATACCAC	KU254567.1

### In vivo injection experiments of recombinant protein *BF/C2*(*A*,* B*)

In this experiment, 40 cultured grass carp were selected for recombinant protein injection and randomly divided into two groups. The experimental group was injected with recombinant protein *BF/C2(A, B)* (concentration 480 ug/ml, injection volume 100 ul/g), and the control group was injected with PBS. After 24 h of injection, five grass carp were randomly selected from the experimental group and the control group. After anaesthesia with MS-222 (25 mg/L) (Sigma Aldrich Co., St. Louis, USA) prior to sample collection. Head-kidney, kidney, liver and spleen were collected and stored at -80 ℃. The total RNA of each tissue was extracted and reverse-transcribed into cDNA. As mentioned above, mRNA expression levels of genes were detected by SYBR green fluorescent qPCR. Table 1 lists the primers used in this study.

### Statistical analysis

All data are indicated as mean ± standard deviation (*N* = 3 or 5) and were analyzed with Statistical Package for Social Sciences Version 25.0 (SPSS Inc., Chicago, IL, USA). A two-sample Student t test was used for the comparisons between groups. Multiple group comparisons were executed by one-way ANOVA and by a Tukey multiple group comparison test. A *p* value < 0.05 was considered as a statistically significant difference.

## Results

### *BF/C2*(*A*, *B*) protein-GCRV-CIK cell lines co-incubation experiment

**Fig. 1:**
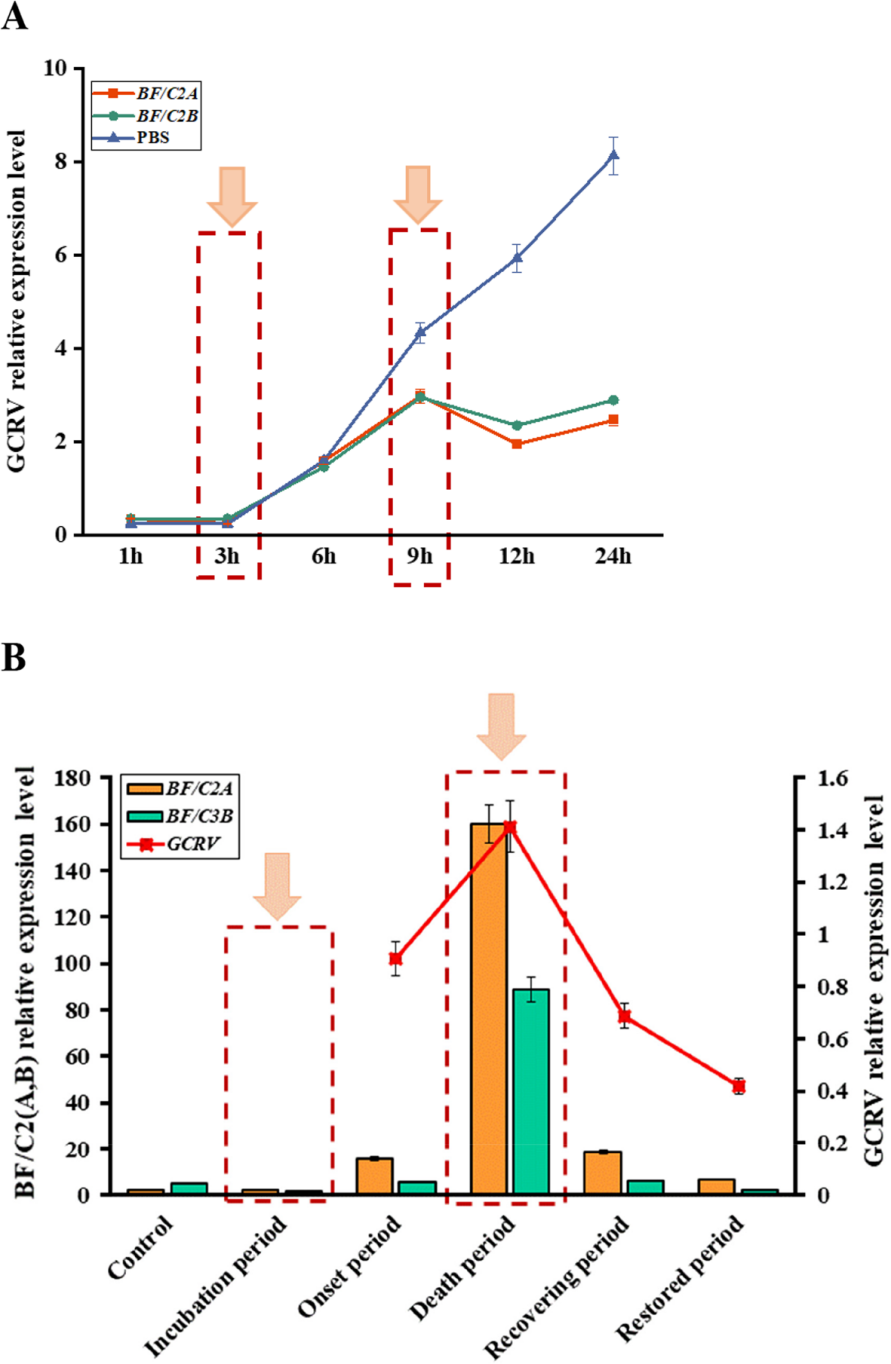
**A** The mRNA expressions of *VP7* relative expression level in CIK. **B** The mRNA expressions of *BF/C2*(*A*, *B*) and *GCRV II* in liver tissues of *C. idella*

The recombinant proteins *BF/C2*(*A*,*B*) were added to CIK cells respectively for 2 h, and then GCRV virus was added and collected for 1 h, 3 h, 6 h, 9 h, 12 h and 24 h cells and RNA extraction was performed. The results of qPCR showed that the recombinant protein *BF/C2*(*A*,*B*) can suppress the virus GCRV relative expression. At 1 h-6 h, the viral replication of GCRV supplemented with *BF/C2A* and *BF/C2B* proteins was not significantly inhibited compared with PBS, and at 6 h-24 h, the viral replication of GCRV supplemented with *BF/C2A* and *BF/C2B* proteins was significantly inhibited compared with PBS. Moreover, the viral replication of GCRV with *BF/C2A* and *BF/C2B* proteins reached its peak at 9 h (Fig. 1A).

### The fold changes in BF/C2(A, B) mRNA expression during GCRV infection

To investigate the dynamic changes in *BF/C2A* and *BF/C2B* during GCRV infection, its mRNA expression levels in the liver of *C. idella* after GCRV challenge at incubation period, onset period, death period, recovering period and restored period were characterized by qPCR. Compared with the control group, the mRNA expression of *BF/C2A* decreased in incubation period, and then showed a trend of first increasing and then decreasing in onset period, death period, recovering period and restored period. It peaked during the death period (Fig. 1B). Compared with the control group, the mRNA expression of *BF/C2B* decreased in incubation period, and then showed a trend of first increasing and then decreasing in onset period, death period, recovering period and restored period. It peaked during the death period (Fig. 1B). During this process, the virus replication volume of GCRV shows a trend of first increasing and then decreasing from onset period, death period, recovering period and restored period, in which the peak is reached in death period (Fig. 1B). The results showed that the mRNA expression levels of *BF/C2A* and *BF/C2B* were the highest when the viral replication of GCRV reached its peak, which further confirmed that *BF/C2*(*A*,* B*) played an important role in the response of grass carp to GCRV. Combined with the results of 2.1 experiment, we believed that 3 h and 9 h of the incubation challenge experiment were equivalent to the incubation period and death period of the live challenge experiment. Therefore, 3 h and 9 h were selected as nodes for subsequent *BF/C2*(*A*,* B*)-treated transcriptome analysis of grass carp kidney cells.

### Sequencing data quality assessment

Through the statistical data of sequencing results (BioProject ID: PRJNA1110017, https://www.ncbi.nlm.nih.gov/sra/?term=PRJNA1110017), a total of 848,131,402 raw reads were obtained, and 818,834,098 clean data were screened. The error rate was 0.01. The percentage of Q20 bases and Q30 bases were greater than 98.67% and 96.48%, respectively, and the GC content was 45.37 ~ 47.49% (Table 2). To obtain mapping data (reads), clean data (reads) are compared with reference genomes (NCBI: gcf_019924925_1_hzgc01, Sex: female). The results are shown in Table 3, and the total mapped from 92.02% to 94.25%. The above results show that Illumina sequencing data and reference genome data are authentic and reliable, and can be used for follow-up studies.

**Table 2:** Statistics reads of transcriptomic sequences

Sample	Raw reads	Clean reads	Error rate (%)	Q20 (%)	Q30 (%)	GC pct (%)
BFC2A_3_1	50,415,090	47,845,632	0.01	98.79	96.8	46.72
BFC2A_3_2	46,165,010	44,351,558	0.01	98.71	96.64	46.09
BFC2A_3_3	45,963,760	44,660,942	0.01	98.86	96.85	46.67
BFC2A_9_1	45,334,986	43,843,892	0.01	98.67	96.49	46.69
BFC2A_9_2	47,319,094	46,030,432	0.01	98.81	96.76	46.52
BFC2A_9_3	45,817,614	43,973,958	0.01	98.88	96.85	47.12
BFC2B_3_1	52,837,114	50,910,212	0.01	98.89	96.88	46.78
BFC2B_3_2	46,831,464	45,484,434	0.01	98.71	96.48	45.37
BFC2B_3_3	53,740,620	51,445,548	0.01	98.82	96.71	46.21
BFC2B_9_1	41,706,454	39,926,932	0.01	98.83	96.55	47.49
BFC2B_9_2	44,453,970	43,190,480	0.01	98.83	96.73	46.67
BFC2B_9_3	47,756,364	46,017,072	0.01	98.89	96.92	46.89
PBS_3_1	46,027,728	44,573,888	0.01	98.8	96.65	46.71
PBS_3_2	43,543,770	42,391,492	0.01	98.7	96.48	46
PBS_3_3	50,137,838	48,520,650	0.01	98.97	97.04	45.99
PBS_9_1	46,715,584	45,444,822	0.01	98.8	96.73	46.74
PBS_9_2	43,335,684	41,920,350	0.01	98.84	96.76	46.78
PBS_9_3	50,029,258	48,301,804	0.01	98.82	96.71	46.42

**Table 3:** Comparison of reads and reference genome

Total mapped reads	Total mapped (%)	Multiple mapped (%)	Exon (%)	Intergenic (%)	Intron (%)
45,049,478	94.16%	4.31%	95.48%	2.15%	2.38%
40,814,351	92.02%	4.32%	94.63%	2.55%	2.82%
41,717,822	93.41%	3.77%	93.92%	2.09%	4.00%
41,047,664	93.62%	4.49%	95.42%	2.47%	2.11%
43,048,097	93.52%	3.82%	95.33%	2.09%	2.58%
40,626,962	92.39%	4.33%	91.00%	3.47%	5.53%
47,490,294	93.28%	4.01%	94.19%	2.11%	3.70%
42,361,782	93.13%	4.21%	92.52%	3.32%	4.15%
47,812,020	92.94%	3.70%	95.26%	2.07%	2.67%
37,630,338	94.25%	4.29%	95.59%	2.17%	2.24%
40,314,874	93.34%	4.04%	94.99%	2.24%	2.77%
43,153,609	93.78%	4.54%	95.37%	2.35%	2.27%
41,562,713	93.24%	4.28%	95.24%	2.29%	2.46%
39,535,145	93.26%	4.12%	95.04%	2.31%	2.65%
45,096,006	92.94%	3.91%	93.68%	2.32%	4.00%
42,697,793	93.96%	3.93%	95.20%	2.11%	2.69%
39,240,491	93.61%	3.95%	95.30%	1.97%	2.73%
45,046,078	93.26%	4.21%	95.38%	2.26%	2.36%

### DEGs analysis

**Fig. 2:**
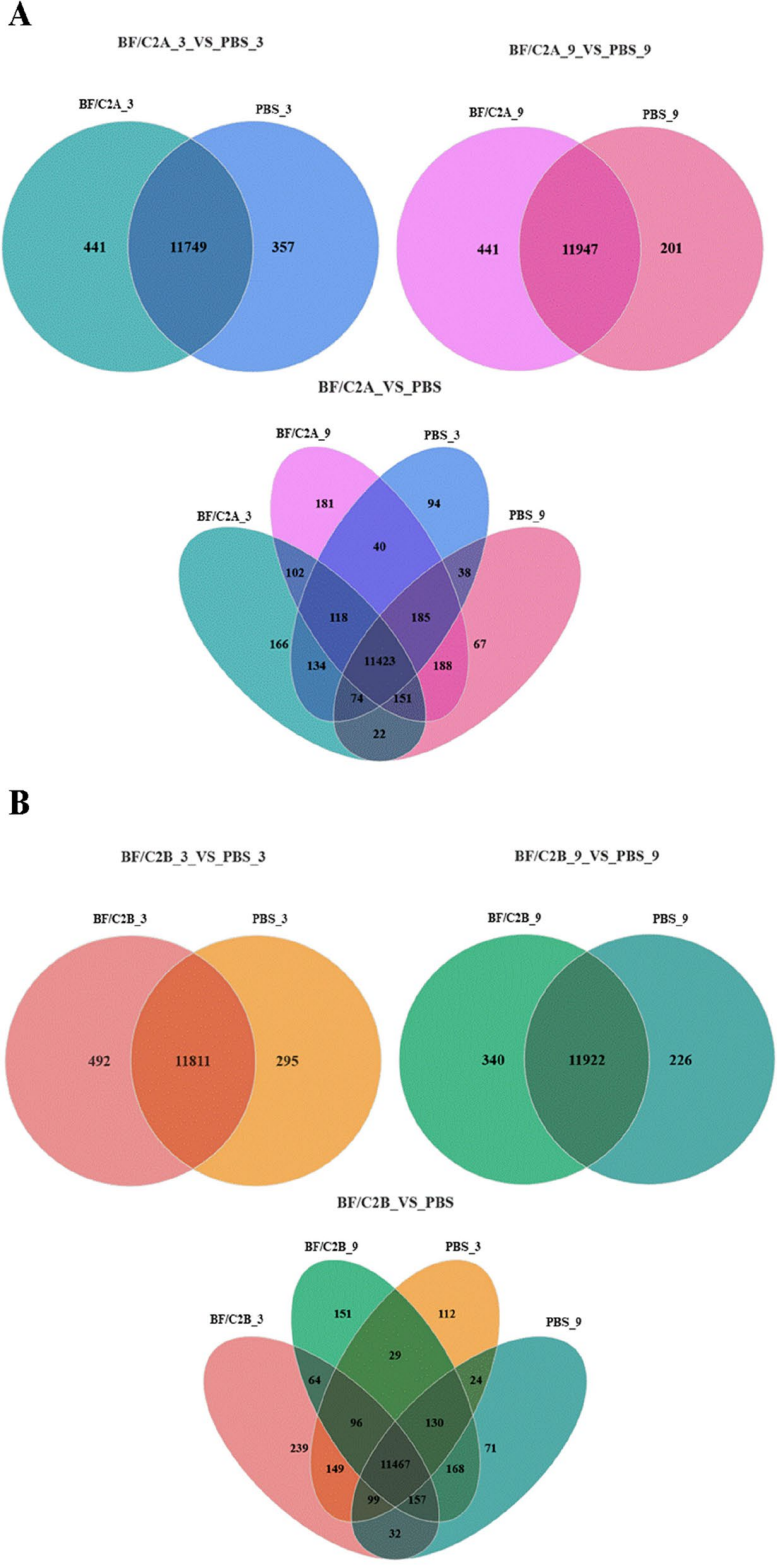
Statistical Venn diagram of DEGs between *BF/C2* groups and PBS groups. **A** The *BF/C2A* group was compared with the PBS group in the three-hour and nine-hour DEGs statistical Venn diagrams; **B** The *BF/C2B* group was compared with the PBS group in the three-hour and nine-hour DEGs statistical Venn diagrams. Note: Circles with different colors represent different gene sets, and numerical values represent the number of genes/transcripts common and unique among different gene sets

**Fig. 3:**
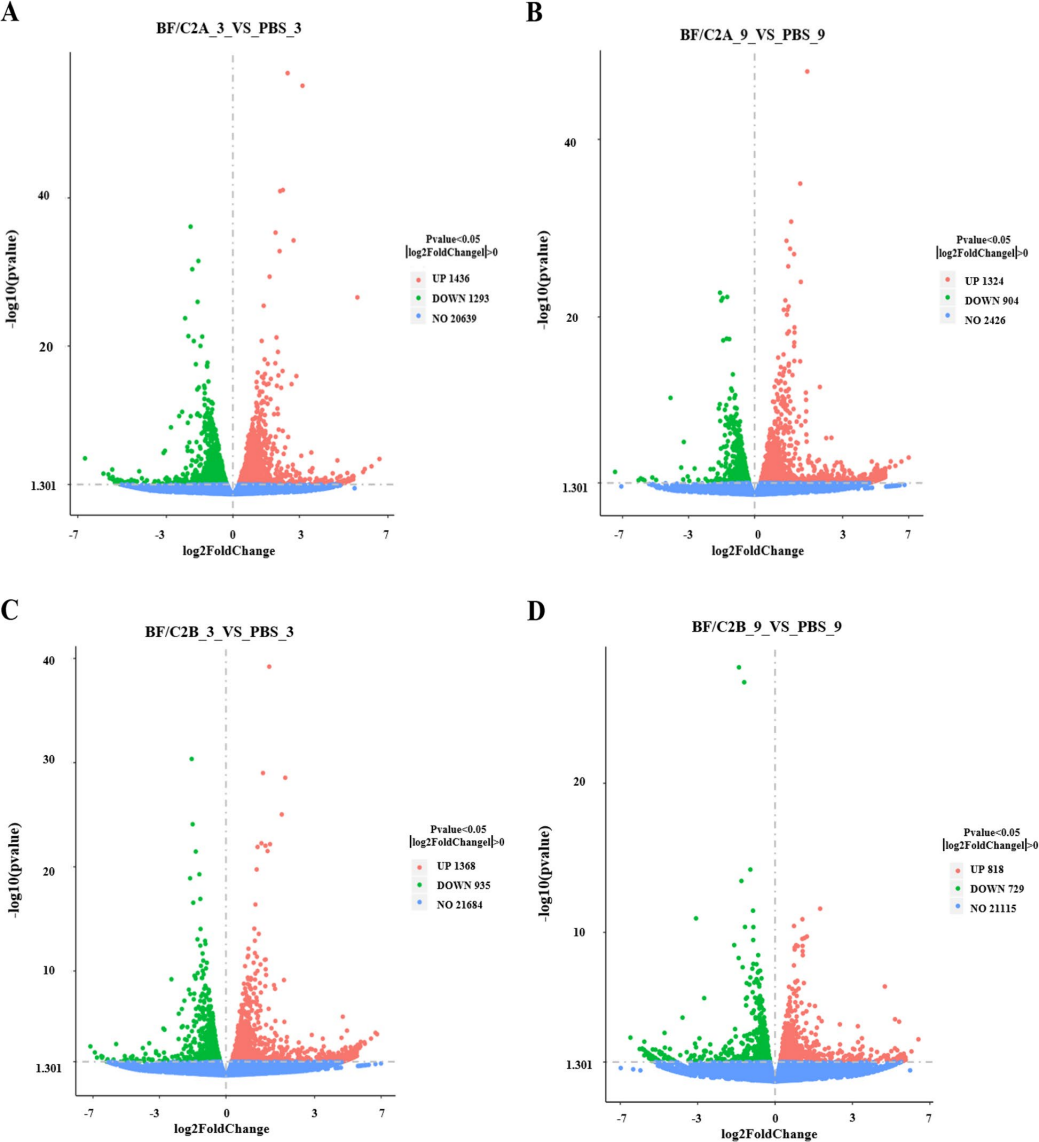
Volcanic map of DEGs between *BF/C2* groups and PBS groups. **A** BF/C2A_3_VS_PBS_3; **B** BF/C2A_9_VS_PBS_9; **C** BF/C2B_3_VS_PBS_3; **D** BF/C2B_9_VS_PBS_9; Note: Each point in the figure represents a specific gene, red dots and green dots represent significantly up-regulated and down-regulated genes, and gray dots are non-significantly different genes

Data analysis showed that BF/C2A_3 group and PBS_3 group shared 11,749 genes, BF/C2A_9 group and PBS_9 group shared 11,947 genes, BF/C2A_3 group, PBS_3 group, BF/C2A_9 group and PBS_9 group shared 11,423 genes (Fig. 2A). BF/C2B_3 group shared 11,811 genes with PBS_3 group, BF/C2B_9 group shared 11,922 genes with PBS_9 group, and BF/C2B_3 group, PBS_3, BF/C2B_9 group and PBS_9 group shared 11,467 genes (Fig. 2B). By screening for the DEGs in these shared genes, compared with PBS_3 group, there were 2729 (up:1436, down:1293) DEGs in BF/C2A_3 group and 2303 (up:1368, down: 935) DEGs in BF/C2B_3 group, respectively. Compared with PBS_9 group, BF/C2A_9 group and BF/C2B_9 group had 2228 (up:1324, down: 904) DEGs and 1547 (up:818, down: 729) DEGs, respectively (Fig. 3A, B; Attachments).

### GO

**Fig. 4:**
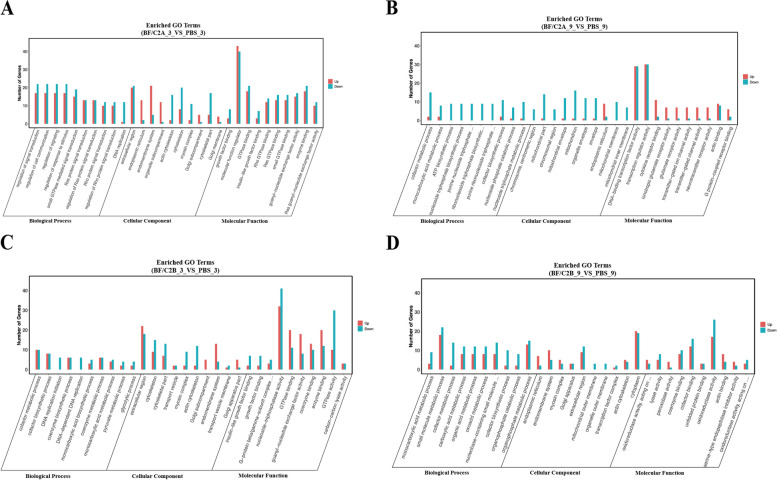
GO classification statistics column chart of DEGs between *BF/C2* groups and PBS groups. **A** BF/C2A_3_VS_PBS_3; **B** BF/C2A_9_VS_PBS_9; **C** BF/C2B_3 _VS_PBS_3; **D** BF/C2B_9_VS_PBS_9; Note: Histogram of GO classification statistics (multiple gene sets): in the graph, the abscissa indicates the secondary classification and terminology of GO, the ordinate indicates the number of genes in the secondary classification

GO database contains three categories: biological process, cellular component, and molecular function, and each category contains several secondary classifications. The top 10 significantly enriched pathways of each categories were analyzed. BF/C2A_3_VS_PBS_3: biological process (DNA replication, Ras protein signal transduction and so on), cellular component (actin cytoskeleton, cytoskeletal part and so on), molecular function(enzyme binding, growth factor binding and so on) (Fig. 4A). BF/C2A_9_VS_PBS_9: biological process (ATP biosynthetic process, cofactor biosynthetic process and so on), cellular component (chromosomal region, chromosome and so on), molecular function (actin binding, cytokine receptor binding and so on) (Fig. 4B). BF/C2B_3_VS_PBS_3: biological process (coenzyme biosynthetic process, coenzyme metabolic process and so on), cellular component (actin cytoskeleton, cytoskeletal part and so on), molecular function(carbon–carbon lyase activity, coenzyme binding and so on) (Fig. 4C). BF/C2B_9_VS_PBS_9: biological process (carboxylic acid metabolic process, cofactor biosynthetic process and so on), cellular component (actin cytoskeleton, cytoplasm and so on), molecular function (actin binding, coenzyme binding and so on) (Fig. 4D).

It is found that BF/C2A_3_VS_PBS_3 and BF/C2A_9_VS_ PBS_9 share one GO signaling pathway: endoplasmic reticulum (cellular component) and BF/C2B_3_VS_PBS_3 and BF/C2B_9_VS_PBS_9 share GO signaling eight pathways: cofactor biosynthetic process, cofactor metabolic process and monocarboxylic acid metabolic process (biological process), actin cytoskeleton, endomembrane system, extracellular region and myosin complex (cellular component), and coenzyme binding (molecular function) (Fig. 4A, B, C, D).

### KEGG functional annotation and enrichment analysis of DEGs

**Fig. 5:**
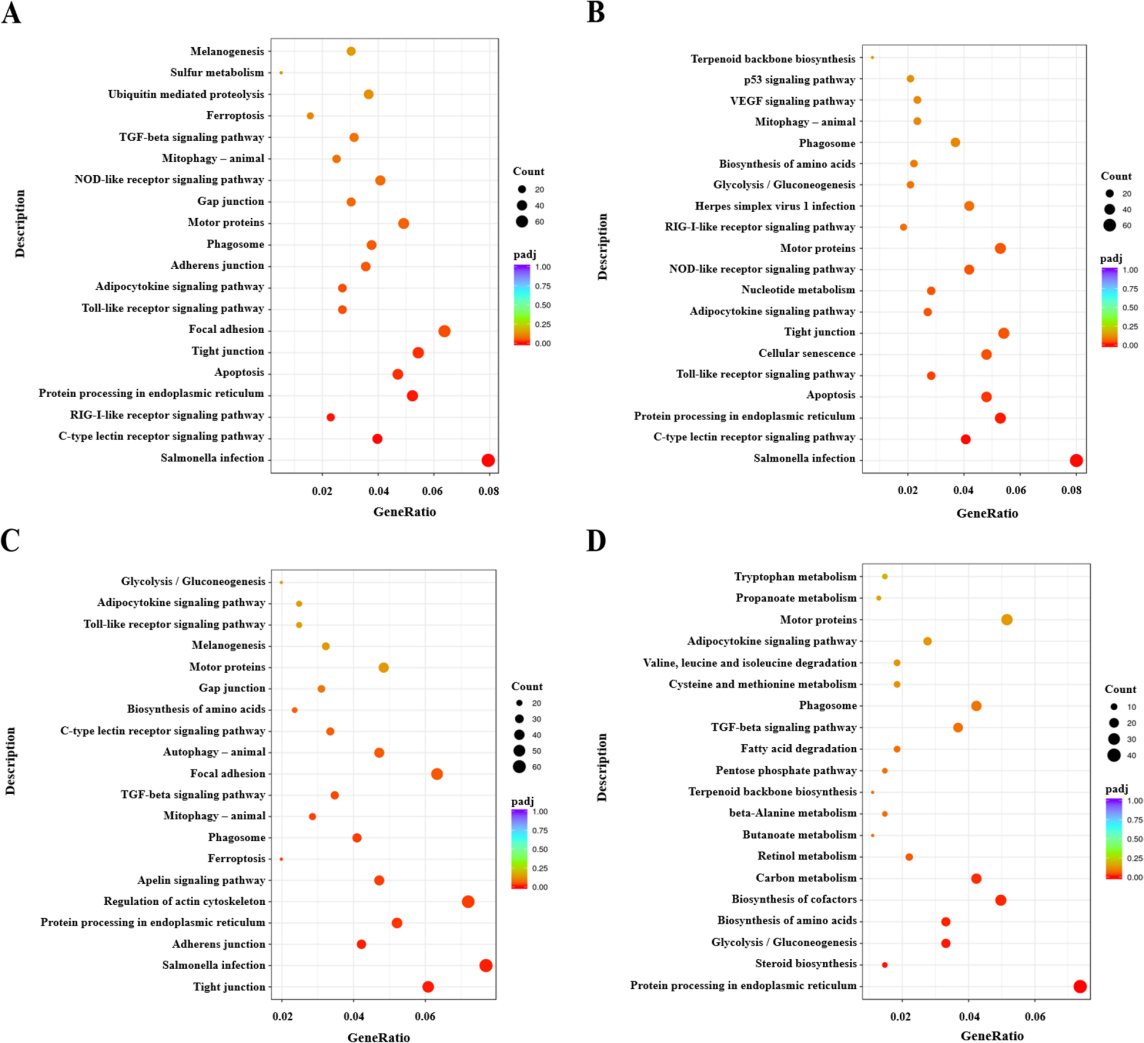
KEGG signal pathway enrichment analysis bubble diagram of DEGs between *BF/C2* groups and PBS groups. **A** BF/C2A_3_VS_PBS_3; **B** BF/C2A_9_VS_PBS_9; **C** BF/C2B_3_VS_ PBS_3; **D** BF/C2B_9_VS_PBS_9. Note: The vertical line is KEGG signaling pathway, and horizontal line represents Rich factor. (The ratio of Sample number to Background number in this term. The greater the Rich factor, the greater the degree of enrichment.) The size of the dot indicates the number of genes in this term, and the color of the dot corresponds to different p-adjust

The DEGs were annotated by KEGG signal pathway using KEGG database. Through the analysis of the top 20 pathways that were significantly enriched. BF/C2A_3_VS_PBS_3: Salmonella infection (76 DEGs), C-type lectin receptor signaling pathway (38 DEGs), RIG-I-like receptor signaling pathway (22 DEGs), Protein processing in endoplasmic reticulum (50 DEGs), Apoptosis (45 DEGs), Tight junction (52 DEGs), Focal adhesion (61 DEGs), Toll-like receptor signaling pathway (26 DEGs), Adipocytokine signaling pathway (26 DEGs), Adherens junction (34 DEGs), Phagosome (36 DEGs), Motor proteins (47 DEGs), Gap junction (29 DEGs), NOD-like receptor signaling pathway (39 DEGs), Mitophagy-animal (24 DEGs), TGF-beta signaling pathway (30 DEGs), Ferroptosis (15 DEGs), Ubiquitin mediated proteolysis (35 DEGs), Sulfur metabolism (5 DEGs) and Melanogenesis (29 DEGs) (Fig. 5A). BF/C2A_9_VS_PBS_9: Salmonella infection (65 DEGs), C-type lectin receptor signaling pathway (33 DEGs), Protein processing in endoplasmic reticulum (43 DEGs), Apoptosis (39 DEGs), Toll-like receptor signaling pathway (23 DEGs), Cellular senescence (39 DEGs), Tight junction (44 DEGs), Adipocytokine signaling pathway (22 DEGs), Nucleotide metabolism (23 DEGs), NOD-like receptor signaling pathway (34 DEGs), Motor proteins (43 DEGs), RIG-I-like receptor signaling pathway (15 DEGs), Herpes simplex virus 1 infection (34 DEGs), Glycolysis / Gluconeogenesis (17 DEGs), Biosynthesis of amino acids (18 DEGs), Phagosome (30 DEGs), Mitophagy–animal (19 DEGs), VEGF signaling pathway (19 DEGs), p53 signaling pathway (17 DEGs) and Terpenoid backbone biosynthesis (6 DEGs) (Fig. 5B). BF/C2B_3_VS_PBS_3: Tight junction (49 DEGs), Salmonella infection (62 DEGs), Adherens junction (34 DEGs), Protein processing in endoplasmic reticulum (42 DEGs), Regulation of actin cytoskeleton (58 DEGs), Apelin signaling pathway (38 DEGs), Ferroptosis (16 DEGs), Phagosome (33 DEGs), Mitophagy– animal (23 DEGs), TGF-beta signaling pathway (28 DEGs), Focal adhesion (51 DEGs), Autophagy–animal (38 DEGs), C-type lectin receptor signaling pathway (27 DEGs), Biosynthesis of amino acids (19 DEGs), Gap junction (25 DEGs), Motor proteins (39 DEGs), Melanogenesis (26 DEGs), Toll-like receptor signaling pathway (20 DEGs), Adipocytokine signaling pathway (20 DEGs) and Glycolysis / Gluconeogenesis (16 DEGs) (Fig. 5C). BF/C2B_9_VS_PBS_9: Protein processing in endoplasmic reticulum (40 DEGs), Steroid biosynthesis (8 DEGs), Glycolysis / Gluconeogenesis (18 DEGs), Biosynthesis of amino acids (18 DEGs), Biosynthesis of cofactors (27 DEGs), Carbon metabolism (23 DEGs), Retinol metabolism (12 DEGs), Butanoate metabolism (6 DEGs), beta-Alanine metabolism (8 DEGs), Terpenoid backbone biosynthesis (6 DEGs), Pentose phosphate pathway (8 DEGs), Fatty acid degradation (10 DEGs), TGF-beta signaling pathway (20 DEGs), Phagosome (23 DEGs), Cysteine and methionine metabolism (10 DEGs), Valine, leucine and isoleucine degradation (10 DEGs), Adipocytokine signaling pathway (15 DEGs), Motor proteins (28 DEGs), Propanoate metabolism (7 DEGs) and Tryptophan metabolism (8 DEGs) (Fig. 5D).

It is found that BF/C2A_3_VS_PBS_3 and BF/C2A_9_VS_ PBS_9 share seven KEGG signaling pathways: C-type lectin receptor signaling pathway, Protein processing in endoplasmic reticulum, Toll-like receptor signaling pathway, Salmonella infection, Apoptosis, Tight junction and Adipocytokine signaling pathway and BF/C2B_3_VS_PBS_3 and BF/C2B_9_VS_PBS_9 share KEGG signaling three pathways: Protein processing in endoplasmic reticulum, Glycolysis / Gluconeogenesis, Biosynthesis of amino acids. BF/C2A_(3, 9)_VS_PBS_(3, 9) and BF/C2B_(3, 9)_VS_PBS_(3, 9) share KEGG signaling pathway: Protein processing in endoplasmic reticulum (Fig. 5).

### DEGs screening

According to the results of differential genes and KEGG enrichment signaling pathway, the shared genes in BF/C2A_3_VS_PBS_3 and BF/C2A_9_VS_PBS_9 shared pathways were statistically analyzed. A total of 144 differential genes were screened out, including 24 (up:19, down:5) genes in C-type lectin receptor signaling pathway, 18 (up:15, down:3) genes in Protein processing in endoplasmic reticulum,16 (up:14, down:2) differential genes in Toll-like receptor signaling pathway, 29 (up:12, down:17) differential genes in Salmonella infection, 22 (up:8, down:14) differential genes in Apoptosis, 20 (up:4, down:16) differential genes in Tight junction and 16 (up:10, down:6) differential genes in Adipocytokine signaling pathway. The shared genes in BF/C2B_3_VS_PBS_3 and BF/C2B_9_VS_PBS_9 shared pathways were statistically analyzed. A total of 20 differential genes were screened out, including 20 (up:17, down:3) genes in Protein processing in endoplasmic reticulum (Table 4).

**Table 4:** Assemble the functional annotation summary of the transcript in the KEGG

Group	KEGG id	Description	Up id	Down id
BFC2A_(3,9) VS PBS_(3,9)	dre04625	C-type lectin receptor signaling pathway	127,524,547/127503933/127498579/127520202/127513496/127496630/127496146/127502947/127515227/127495036/127525667/127515912/127512473/127522689/127498959/127517920/127525380/127520732/127494950/127502124/127521489/127519975/127513816/127504966/127510537/127517001	127,502,688/127517087/127501241/127508034/127503188/127522287/127524770
	dre04141	Protein processing in endoplasmic reticulum	127,510,502/127521597/127502859/127505798/127494786/127518872/127518675/127517703/127499346/127502050/127516737/127512731/127514772/127504645/127522873	127,498,555/127503188/127508471
	dre04620	Toll-like receptor signaling pathway	127,511,914/127525235/127513496/127520202/127515912/127511466/127498579/127525380/127507772/127525667/127523422/127502947/127499039/127524669	127,502,688/127503188
	dre05132	Salmonella infection	127,498,579/127520202/127513496/127511914/127502947/127525667/127515912/127525380/127507772/127522694/127497953/127503263	127,520,112/127502688/127514626/127504795/127509863/127504593/127517491/127515318/127508367/127506178/127525720/127508282/127517989/127522074/127503188/127504771/127506414
	dre04210	Apoptosis	127,498,579/127513496/127502947/127525667/127496831/127515912/127525380/127503263	127,498,920/127520112/127502688/127514626/127501495/127508471/127509863/127504593/127508367/127497888/127517989/127521895/127503188/127502321
	dre04530	Tight junction	127,509,857/127503957/127499766/127496895	127,520,112/127521140/127502688/127514626/127504795/127509863/127504593/127503506/127516769/12750836/127503508/127517989/127523283/127522074/127503188/127517995
	dre04920	Adipocytokine signaling pathway	127,498,579/127502110/127502947/127525667/127500028/127515912/127522307/127513515/127503930/127525380	127,508,288/127514171/127506370/127512758/127503188/127503522
BFC2B_(3,9) VS PBS_(3,9)	dre04141	Protein processing in endoplasmic reticulum	127,521,597/127502859/127510502/127494786/127498391/127494833/127518872/127505798/127517703/127506379/127518675/127520848/127512586/127502050/127512730/127499346/127524532	127,498,555/127508471/127503188

### qPCR verification

**Fig. 6:**
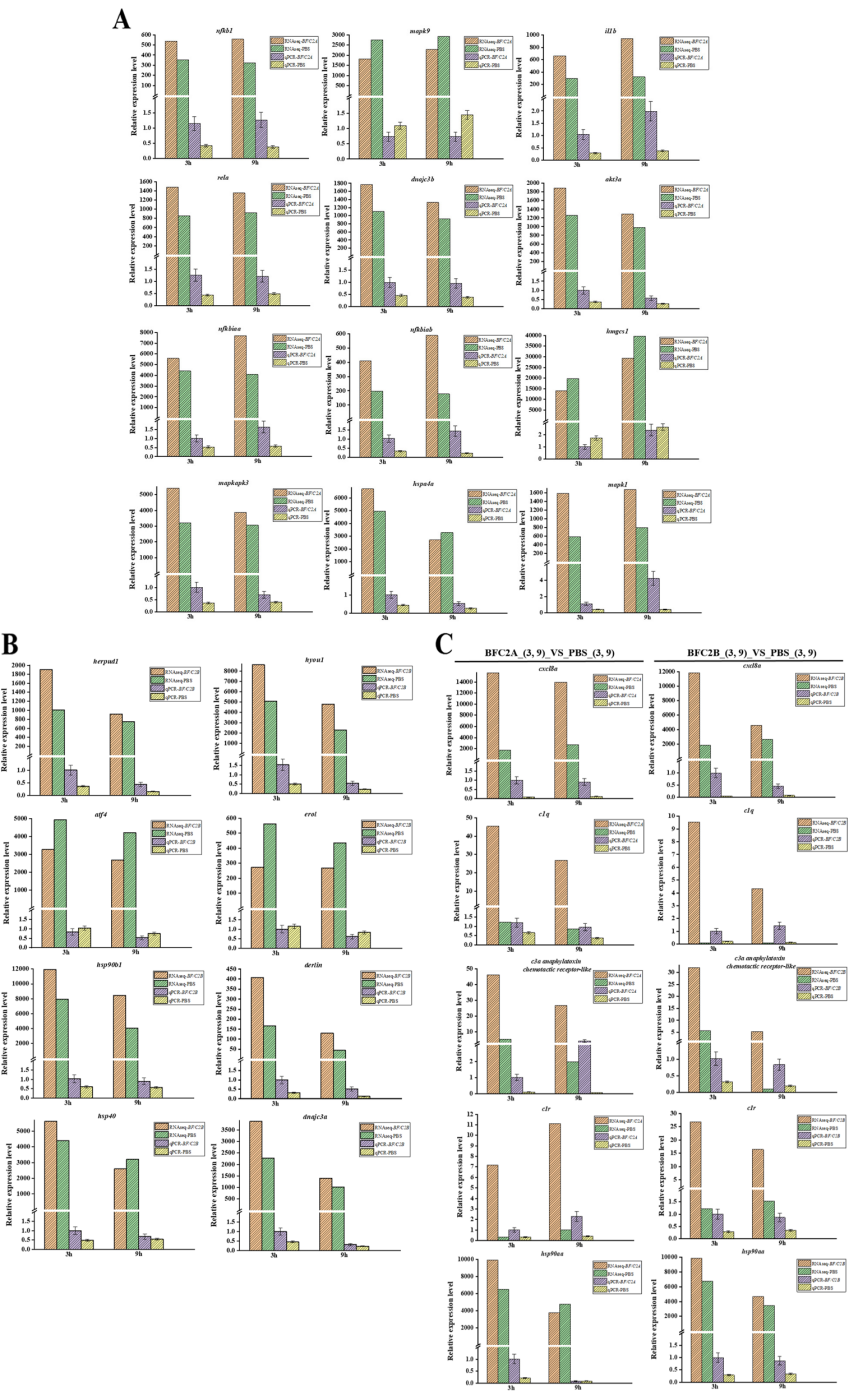
Validation of the expression of 25 genes in transcriptome data by qPCR. *β*-Actin was used as a reference gene. There are three duplicates for each sample. **A** The 12 DEGs of BFC2A_(3, 9)_VS_PBS_(3, 9); **B** The 8 DEGs of BFC2B_(3, 9)_VS_PBS_(3, 9); **C** The 5 DEGs shared by BFC2A_(3, 9)_VS_PBS_(3, 9) and BFC2A_(3, 9)_VS_PBS_(3, 9)

To verify the reliability of high-throughput sequencing results, 25 DEGs were expressed by qPCR. These 25 differential genes include 12 DEGs of BF/C2A_(3, 9)_VS_PBS_(3, 9): *mapk1* (mitogen-activated protein kinase 1), *il1b* (interleukin 1, beta), *rela* (v-rel avian reticuloendotheliosis viral oncogene homolog A), *nfkbiab* (nuclear factor of kappa light polypeptide gene enhancer in B-cells inhibitor, alpha b), *akt3a* (v-akt murine thymoma viral oncogene homolog 3a), *nfkb1* (nuclear factor of kappa light polypeptide gene enhancer in B-cells 1), *nfkbiaa* (nuclear factor of kappa light polypeptide gene enhancer in B-cells inhibitor, alpha a), *mapk9* (mitogen-activated protein kinase 9), *dnajc3b* (DnaJ (Hsp40) homolog, subfamily C, member 3b), *hmgcs1* (3-hydroxy-3-methylglutaryl-CoA synthase 1), *mapkapk3* (mitogen-activated protein kinase 3), *hspa4a* (heat shock protein 4a). The 8 DEGs of BF/C2B_(3, 9)_VS_PBS_(3, 9): *hyou1* (hypoxia up-regulated 1), *hsp90b1* (heat shock protein 90, beta (grp94), member 1), *dnajc3a* (DnaJ (Hsp40) homolog, subfamily C, member 3a), *herpud1* (homocysteine-inducible, endoplasmic reticulum stress-inducible, ubiquitin-like domain member 1), *erol* (endoplasmic reticulum oxidoreductase 1 alpha), *atf4* (atf4b—activating transcription factor 4b), *hsp40* (heat shock protein 40), *derlin* (derlin). And the 5 DEGs shared by BF/C2A_(3, 9)_VS_PBS_(3, 9) and BF/C2B_(3, 9)_VS_PBS_(3, 9): *C3a anaphylatoxin chemotactic receptor-like*, *C1r* (complement C1r), *C1q* (complement C1q), *hspa5* (heat shock protein 5), *hsp90aa1.2* (heat shock protein 90, alpha (cytosolic), class A member 1, tandem duplicate 2), *cxcl8a* (chemokine (C-X-C motif) ligand 8a) (Table 5). The results showed that the expression levels of these genes were consistent with the transcriptome expression analysis (Fig. 6A,B,C).

**Table 5:** DEGs information description

Name	Gene id	Description	Change
*mapk1*	127,513,496	C-type lectin receptor signaling pathway	Up
		Toll-like receptor signaling pathway	
		Salmonella infection	
		Apoptosis	
*il1b*	127,520,202	C-type lectin receptor signaling pathway	Up
		Toll-like receptor signaling pathway	
		Salmonella infection	
*rela*	127,515,912	C-type lectin receptor signaling pathway	Up
		Toll-like receptor signaling pathway	
		Salmonella infection	
		Apoptosis	
		Adipocytokine signaling pathway	
*nfkbiaa*	127,502,947	C-type lectin receptor signaling pathway	Up
		Toll-like receptor signaling pathway	
		Salmonella infection	
		Apoptosis	
		Adipocytokine signaling pathway	
*nfkbiab*	127,498,579	C-type lectin receptor signaling pathway	Up
		Toll-like receptor signaling pathway	
		Salmonella infection	
		Apoptosis	
		Adipocytokine signaling pathway	
*nfkb1*	127,525,667	C-type lectin receptor signaling pathway	Up
		Toll-like receptor signaling pathway	
		Salmonella infection	
		Apoptosis	
		Adipocytokine signaling pathway	
*akt3a*	127,525,380	C-type lectin receptor signaling pathway	Up
		Toll-like receptor signaling pathway	
		Salmonella infection	
		Apoptosis	
		Adipocytokine signaling pathway	
*mapk9*	127,503,188	C-type lectin receptor signaling pathway	Down
		Toll-like receptor signaling pathway	
		Salmonella infection	
		Apoptosis	
		Adipocytokine signaling pathway	
		Protein processing in endoplasmic reticulum	
		Tight junction	
*dnajc3b*	127,516,821	Protein processing in endoplasmic reticulum	Up
*hmgcs1*	127,520,922	Valine, leucine and isoleucine degradation	Up
		Terpenoid backbone biosynthesis	
		Butanoate metabolism	
		PPAR signaling pathway	
*mapkapk3*	127,522,641	C-type lectin receptor signaling pathway	Up
		Toll-like receptor signaling pathway	
		Salmonella infection	
		Apoptosis	
		Adipocytokine signaling pathway	
		Protein processing in endoplasmic reticulum	
		Tight junction	
*hspa4a*	127,503,962	Tight junction	Up
*hyou1*	127,521,597	Protein processing in endoplasmic reticulum	Up
*hsp90b1*	127,510,502	Protein processing in endoplasmic reticulum	Up
*dnajc3a*	127,518,675	Protein processing in endoplasmic reticulum	Up
*herpud1*	127,499,346	Protein processing in endoplasmic reticulum	Up
*erol*	127,498,555	Protein processing in endoplasmic reticulum	Down
*atf4*	127,508,471	Protein processing in endoplasmic reticulum	Down
*hsp40*	127,518,872	Protein processing in endoplasmic reticulum	Up
*derlin*	127,517,703	Protein processing in endoplasmic reticulum	Up
*c3a anaphylatoxin chemotactic receptor-like*	127,501,507	Complement system	Up
*c1r*	127,497,057	Complement system	Up
*c1q*	127,508,669	Complement system	Up
*hsp90aa1.2*	127,501,922	Protein processing in endoplasmic reticulum	Up
*cxcl8a*	127,511,914	Protein processing in endoplasmic reticulum	Up

### In vivo injection experiments of recombinant protein BF/C2(A, B)

**Fig. 7:**
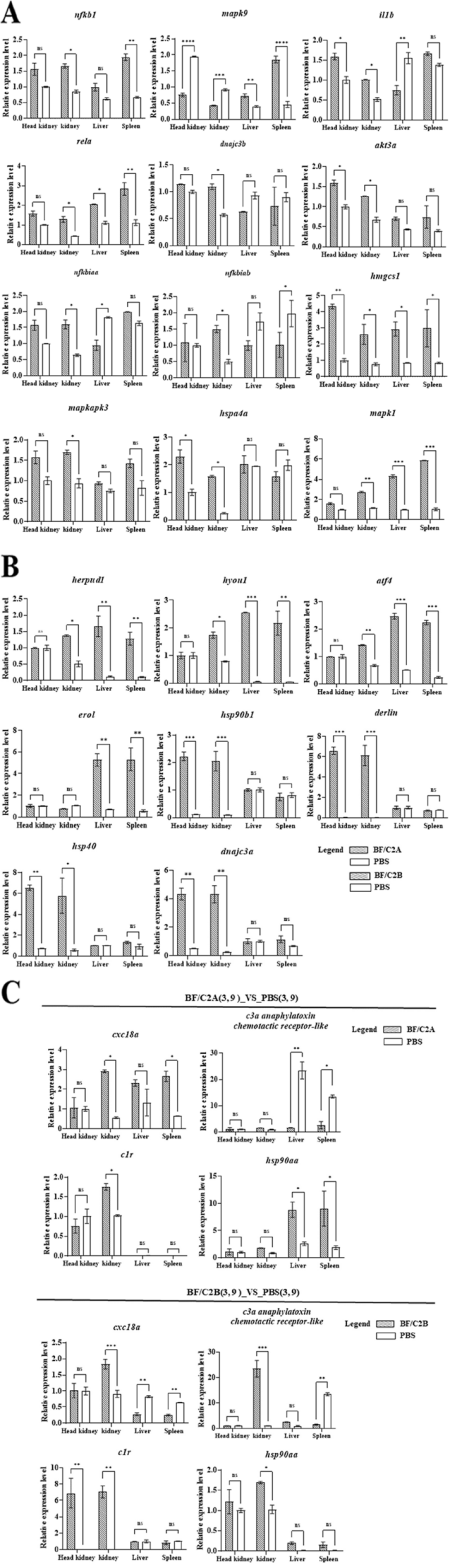
*BF/C2* (**A**, **B**) recombinant protein was injected in vivo for DEGs detection. β-Actin was used as a reference gene. There are three duplicates for each sample **A** The mRNA expressions of the 12 DEGs of BFC2A_(3, 9)_VS_PBS_(3, 9) in head-kidney, kidney, liver and spleen of *C. idella*; **B** The mRNA expressions of the 8 DEGs of BFC2B_(3, 9)_VS_PBS_(3, 9) in head-kidney, kidney spleen and liver of *C. idella*; **C** The mRNA expressions of the 4 DEGs of shared by BFC2A_(3, 9)_VS_PBS_(3, 9) and BFC2B_(3, 9)_VS_PBS_(3, 9) in head-kidney, kidney spleen and liver of *C. idella*

The qPCR analysis was performed on the liver, spleen, kidney and head kidney of the experimental group and the control group. The results showed that the expression trend in these immune tissues was consistent with the trend of transcriptome analysis. This further reflects the importance of these genes (Fig. 7 A,B,C).

## Discussion

*BF/C2*, a critical pathway molecule within the complement system, is essential for its function. Previous studies have explored the impact of *Aeromonas hydrophila* on *BF/C2* in various fish species, including grass carp and *Oryzias latipes* [23]. In grass carp, it was observed that *BF/C2b* transcription is prevalent across different tissues and is induced both in vivo and in vitro by *A. hydrophila*, as well as by lipopolysaccharide and flagellin stimuli [10]. Overexpression of *BF/C2b* in cells led to significantly increased transcription levels of all complement components except *C5*. Following *A. hydrophila* infection or stimulation, notable upregulation was observed in the levels of *BF/C2b*, *IL1β*, *TNF-α*, *IFN*, *CD59*, *C5aR1*, and *ITGβ-2* in grass carps [10, 12]. Conversely, *BF/C2b* transcription was down-regulated in cells interfered with after *A. hydrophila* attack, which also induced the NF-κB signaling pathway, underscoring the crucial role of *BF/C2b* in the innate immunity of grass carp [10, 12]. Additionally, studies have indicated that *BF/C2* also significantly contributes to the response of live and kidney cells to GCRV infection in grass carp. There was a notable increase in *BF/C2* expression in the kidney, liver, spleen, and head kidney cells following GCRV infection [21]. It was also noted that *BF/C2* could activate the complement *C3*, though the exact mechanisms through which grass carp exerts its effects post-*C3* activation by *BF/C2* remain unexplored [21]. Similar to mammals, grass carp utilize components *C3* through *C9* to ultimately form the membrane attack complex (MAC), highlighting functional parallels in immune responses across these species.

CLRs (C-type lectin receptor) as important families of PRRs (pattern recognition receptors), playing essential roles in the innate immunity of fish. They facilitate various immune functions such as microbial agglutination, anti-bacterial or anti-viral responses, cell adhesion, enhanced opsonization, phenoloxidase activation, nodular formation, phagocytosis, and encapsulation [24, 25]. Toll-like receptors (TLRs) are crucial protein molecules in innate immunity, acting as a bridge to adaptive immunity. These single transmembrane non-catalytic proteins recognize conserved molecular structures from microorganisms. Upon breach of physical barriers by pathogens like microorganisms and viruses, TLRs recognize these invaders and trigger immune cell responses [26–28]. Similar to many PRRs, C-type lectins can activate the Toll receptor signaling pathway and the immune deficiency signaling pathway by recognizing pathogen-associated molecular patterns (PAMPs). This activation releases antimicrobial peptides, antiviral factors, and other immune-active substances. It also triggers the prophenoloxidase cascade, leading to melanin and active oxide production, promoting cell melanosis and nodule formation, ultimately completing the immune defense against pathogens [29]. Protein processing in the endoplasmic reticulum was notably enriched in both BF/C2A_(3, 9)_VS_PBS_(3, 9) and BF/C2B_(3, 9)_VS_PBS_(3, 9). The endoplasmic reticulum plays a crucial role in protein synthesis, processing, and modification. During viral infections and calcium homeostasis disturbances, misfolded protein accumulation can lead to severe ER stress. As part of the endoplasmic reticulum stress-mediated pathway, protein processing in the endoplasmic reticulum is a principal pathway for cell apoptosis, interacting with the death receptor pathway and the mitochondrial pathway [30]. These findings underscore the importance of protein processing in the endoplasmic reticulum as a critical pathway for future studies to understand the function of *BF/C2*(*A*, *B*) in grass carp.

We identified 12 significantly different genes in the BF/C2A_(3, 9)_VS_PBS_(3, 9) group, and 8 significantly different genes were identified in the BF/C2B_(3, 9)_VS_PBS_(3, 9) group. Among various intracellular signaling pathways, the *MAPK* cascade is particularly pivotal, with mapk1 and mapkapk3 being crucial components. These proteins are key in translating external stimuli into a broad array of cellular responses, including growth, inflammation, and stress response [31–33]. The regulatory function of the *MAPK* family in human physiology and pathology is a subject of ongoing deep research. Activated *mapk1* can migrate from the cytoplasm to the nucleus, where it influences gene transcription and translation by phosphorylating multiple transcription factors, thus propagating upstream extracellular stimuli to various downstream effector molecules in the nucleus [31–33]. When grass carp kidney cells incubated with *BF/C2A* exhibit increased expression of *mapk1*, further studies are needed to determine if it also undergoes the aforementioned migration and action. *Il1b*, a member of the interleukin-1 cytokine family, is a key pro-inflammatory factor that plays an important role in the body's immune response. It is mainly secreted by sentinel cells of the innate immune system, such as mononuclear macrophages, etc. [34, 35]. *Il1b*, part of the interleukin-1 cytokine family, plays a crucial role as a pro-inflammatory factor in the immune response. It is primarily secreted by sentinel cells of the innate immune system, such as mononuclear macrophages [34, 35]. *Il1b* operates through autocrine, paracrine, and endocrine mechanisms, affecting a range of cells including mononuclear macrophages, fibroblasts, epithelial cells, and endothelial cells [34–37]. The activation of *Il1b* occurs when PAMPs or Damage-Associated Molecular Patterns (DAMPs) are recognized by Pattern Recognition Receptors (PRRs), which include TLRs, NOD-like receptors (NODs), retinoic acid-inducible gene I-like receptors (RLRs), CLRs, and various intracellular DNA receptors [38–40]. These receptors subsequently activate inflammatory transcription factors upon recognizing these molecular patterns. Research indicates that the absence of *Il1b* in mice leads to high susceptibility to group B streptococcus, suggesting a role for *Il1b* in bacterial infection inhibition [41]. Moreover, *Il1b* knockout mice exhibit a higher viral load in the brain compared to wild-type, indicating a reduced immune response [42]. Additionally, *Il1b* has been shown to inhibit the replication of the Human Immunodeficiency Virus type-1 [43]. Beyond its immunomodulatory roles, *Il1b* also promotes cell proliferation and differentiation, as evidenced by studies showing that *Il1b* can stimulate endothelial progenitor cells to form blood vessels through the activation of the MAPK phosphorylation pathway [44]. Nuclear-factor κB (NF-κB) is a ubiquitous transcription factor with multifaceted regulatory roles in cells, participating in a variety of physiological and pathological processes such as inflammation, immune response, oxidative stress, and apoptosis [45–47]. The NF-κB pathway is central to the regulation of various cytokine networks and controls over 200 target genes, most of which are inflammatory genes involved in the inflammatory response. This includes genes for adhesion molecules, interleukins, chemotactic factors, acute phase response genes, and cytokines, such as *IL1b* and tumor necrosis factor (*TNF-α*) [45–47]. *Nfkb1*, *nfkbiab*, *nfkbiaa*, and *rela* are critical members of the NF-κB protein family and play significant roles in these responses. For instance, *nfkb1* encodes the p105 and p50 subunits of the NF-κB family, where p50/p50 homodimers exhibit anti-inflammatory effects by inhibiting the transcription of inflammatory cytokines like *TNF-α* and interleukin-12 (*IL-12*), and promoting the transcription of the anti-inflammatory cytokine interleukin-10 (*IL-10*) [48–51]. The *nfkbia* (*a*, *b*) genes encode *IκBα*, which, in its resting state, binds to p65, masking the nuclear localization signal of the p50 protein and thereby keeping NF-κB inactive in the cytoplasm [48–51]. Upon stimulation by agents such as lipopolysaccharide, reactive oxygen species, or *TNF-α*, *IκBα* becomes phosphorylated and is subsequently ubiquitinated and degraded in the proteasome. This degradation releases the p50/p65 heterodimer, which then rapidly translocates into the nucleus to activate target genes, thereby participating in and regulating a range of physiological and pathological processes [48–51].

Heat shock proteins (*HSPs*) are stress proteins produced by the body in response to external stressors such as heat stress, trauma, infection, tumors, and hypoxia. They are also known as molecular chaperone proteins due to their protective roles in cellular physiology [52]. *HSPs* are categorized based on their molecular weights into six classes: large molecular *HSPs* (100–110 kDa), *HSP90* (83–90 kDa), *HSP70* (66–78 kDa), *HSP60*, *HSP40*, and small molecular *HSPs* (15–30 kDa) [53]. *HSP90b1*, a member of the heat shock protein 90 family, shares 50% homology with *HSP90* and is also referred to as Endoplasmin due to its location in the endoplasmic reticulum cavity, where it acts as a potent molecular adjuvant. In protein synthesis, *HSP90b1* is involved in the correct folding, stretching, assembly, and transport of proteins. It binds to unfolded proteins, prevents protein aggregation, and inhibits the secretion of misfolded proteins. *HSP90b1* has been identified as a potential molecular carrier for tumor antigens, aiding in tumor antigen presentation and activating CD8 + cytotoxic T lymphocytes, which are crucial for anti-tumor specific immune responses. In gastric cancer cells, *HSP90b1* interacts with the client protein *LRP5* and inhibits the ubiquitin–proteasome degradation pathway of *LRP5*, thereby influencing the progression of gastric cancer [54]. Additionally, lncRNA-AC245100.4 binds to *HSP90*, altering its chaperone function, increasing the stability of the client protein IKK, and further promoting the growth of prostate cancer [55].

As a crucial member of the heat shock protein family, the *hsp40* gene plays a significant role in physiological and biochemical processes such as protein translation, folding, and translocation by stimulating the adenosine triphosphatase (ATPase) activity of the *hsp70* gene [56, 57]. The J domain of the *hsp40* gene, represented by *dnajc3a* and *dnajc3b*, collaborates with the *hsp70* gene to regulate various life processes including apoptosis, cell metabolism, and cell survival. This interaction is vital for cellular immune responses, body growth, and embryonic development [58–60]. The *hsp40* gene is ubiquitously present across a wide range of biological cells, from chlamydomonas to mammals [61–64]. In particular, 57 *hsp40* family genes have been identified in *Channel Catfish* [61], 31 in the maternal lineage of Mare [62], 36 in *Corhynchus Mykiss* [63], and 50 in *Japanese Flounder* [64]. In aquatic biology research, numerous members of the heat shock protein family have been shown to be involved in the response mechanisms of aquatic organisms to heat stress. For instance, Bai Xueqiu et al. [65] observed that the relative expression levels of the *hsp70* and *hsp90* genes in various tissues of *Echinus intermedius* were upregulated following high-temperature induction. Similarly, the relative expressions of *hsp40*, *hsp70*, and *hsp90* genes in the gill tissue of Japanese shrimp (*Marsupenaeus japonicus*) were increased after exposure to heat stress at 32℃ [58]. Liu Tianyu et al. [66] performed heat treatments at 28℃ on Chlamys farreri and discovered that such treatments could induce apoptosis in its blood cells, leading to a decrease in the immunity of *C. Farreri*. however, the relative expression of mRNA for the *hsp70* and *hsp90* genes in blood cells was upregulated, thereby helping to protect cells and tissues from damage.

## Conclusions

In summary, based on the results of in vitro and in vivo experiments, 3 h(incubation period) and 9 h(peak period) were selected as the critical points of this study, and a series of differential genes (*mapk1*, *il1b*, *rela*, *nfkbiab*, *akt3a*, *hyou1*, *hsp90b1*, *dnajc3a* et al.) and pathways (C-type lectin receptor signaling pathway, protein processing in the endoplasmic reticulum, Toll-like receptor signaling pathway, Salmonella infection et al.) caused by *BF/C2* in response to GCRV infection were analyzed by transcriptome sequencing. By analyzing these differential genes and pathways, the immune molecules that may be involved in *BF/C2* response to GCRV infection were screened out. The immune mechanism of *BF/C2* against GCRV infection in grass carp was further supported by the mRNA expression changes of these candidate molecules. This study provides a basis for further research on the immune mechanism of *BF/C2* in response to GCRV infection. In addition, we also found that *BF/C2A* and *BF/C2B* may have different immune mechanisms in response to GCRV infection, and this result will be the focus of future research.

### Supplementary Information

Supplementary Material 1.

Supplementary Material 2.

Supplementary Material 3.

Supplementary Material 4.

## Data Availability

All data generated or analyzed during this study are included in this article, and the raw data can be obtained by contacting the corresponding author. The transcriptome sequencing raw data can be found through the National Center for Biotechnology Information (NCBI) database, entry number: (BioSample) PRJNA1110017. (https://www.ncbi.nlm.nih.gov/sra/?term=PRJNA1110017)

## Abbreviations

BF/C2 (A,B): Complement factor BF/C2(A,B)
qRT-PCR: Quantitative real-time PCR
CIK: Kidney cell
GCRV: Grass carp reovirus
DEGs: Differentially expressed genes
